# Metabolic Signatures in Pericardial Fluid and Serum Are Associated with Long-Term Restenosis After Isolated Coronary Artery Bypass Grafting

**DOI:** 10.3390/jcm15093436

**Published:** 2026-04-30

**Authors:** Xiaozheng Zhou, Lin Zheng, Zhiyong Du, Jiyuan Luo, Kun Hua, Xiubin Yang

**Affiliations:** 1 Department of Cardiac Surgery, Beijing Anzhen Hospital, Capital Medical University, Beijing 100054, China; zxzfighting@163.com (X.Z.); zhenglin28@yeah.net (L.Z.); luojiyuanm@126.com (J.L.); 2 Beijing Institute of Heart, Lung and Blood Vessel Diseases, Beijing Anzhen Hospital, Capital Medical University, Beijing 100029, China; duzhiyong1989@163.com

**Keywords:** coronary artery bypass grafting, restenosis prediction, targeted metabolomics, metabolic biomarkers, pericardial fluid, restenosis, risk prediction

## Abstract

**Background/Objectives:** Restenosis following coronary artery bypass grafting (CABG) remains a major long-term complication that adversely affects patient prognosis. Although prior studies have investigated clinical features, imaging parameters, and circulating biomarkers for restenosis risk stratification, the metabolic mechanisms underlying long-term restenosis—particularly those reflecting both the local cardiac microenvironment and systemic circulation—remain poorly defined. Therefore, this study aims to identify restenosis-associated metabolic alterations and develop a risk prediction model based on integrated targeted metabolomic profiling of pericardial fluid (PF) and serum in patients undergoing isolated CABG. **Methods:** Patients undergoing isolated CABG were prospectively enrolled. Paired PF and serum samples were collected during surgery or the perioperative period for targeted metabolomic analysis. Differential metabolite (DM) analysis was performed between patients with and without restenosis. Key metabolites were selected to construct a restenosis risk prediction model, which was subsequently evaluated in training and validation cohorts. **Results:** Compared with patients without restenosis, those who developed restenosis exhibited two key differential metabolites identified in PF and serum: 7α-Hydroxy-4-cholesten-3-one and Phenoxyacetic acid (PAA). A logistic regression-based prediction model incorporating these metabolites was developed and evaluated using receiver operating characteristic (ROC) analysis, integrated discrimination improvement (IDI), and decision curve analysis (DCA). The model demonstrated robust predictive performance in both training and validation cohorts. Kaplan–Meier survival analysis further revealed that higher model scores were significantly associated with an increased risk of long-term restenosis in the training cohort (HR = 1.44, *p* = 0.047) and validation cohort (HR = 1.83, *p* = 0.012). **Conclusions:** This study provides the first evidence that integrated metabolomic signatures derived from PF and serum are associated with long-term restenosis after CABG. By capturing complementary metabolic information from the local cardiac microenvironment and systemic circulation, this integrated approach enhances current understanding of restenosis biology and supports the potential clinical utility of targeted metabolomics for long-term restenosis risk prediction following CABG.

## 1. Introduction

Coronary artery bypass grafting (CABG) remains one of the most effective treatments for advanced coronary artery disease, providing durable improvements in survival and quality of life [1]. Nevertheless, long-term graft restenosis continues to limit the sustained benefit of CABG, with up to 20% of vein grafts developing significant luminal narrowing within ten years after surgery [2,3]. Beyond its clinical consequences, restenosis represents a complex biological process involving vascular remodeling, inflammation, and metabolic dysregulation within grafted vessels and their surrounding cardiac microenvironment [4].

In translational cardiovascular research, increasing attention has been directed toward leveraging molecular signatures—particularly metabolites—to elucidate disease mechanisms while offering predictive and diagnostic value. However, metabolomic investigations of post-CABG restenosis have largely focused on serum or other conventional biofluids. In contrast, the metabolic landscape of the local cardiac microenvironment, particularly pericardial fluid (PF)—a biofluid in direct contact with the heart and bypass grafts—remains underexplored. This limited evaluation of cardiac-adjacent metabolic alterations has constrained a comprehensive understanding of the metabolic perturbations driving graft restenosis.

Over the past decade, metabolomics has emerged as a powerful tool for mapping disease-associated biochemical alterations and capturing clinically meaningful changes in metabolic networks [5]. Biofluid metabolites provide a dynamic snapshot of physiological and pathological processes, making them attractive candidates for biomarker discovery [6]. Although serum-based metabolomic profiling has yielded insights into acute coronary syndromes, it does not fully reflect the specialized cardiac milieu. In contrast, PF—the serous fluid surrounding the heart—closely mirrors the local cardiac microenvironment and may reveal early metabolic changes associated with vascular remodeling and inflammation preceding clinical restenosis [7]. Our previous work validated the predictive value of PF metabolomics in postoperative atrial fibrillation, demonstrating that PF-derived metabolites can serve as robust prognostic biomarkers. Additionally, PF metabolomics has been successfully applied in studies of myocardial infarction [8,9,10]. However, its utility in predicting long-term outcomes and graft restenosis after CABG has not been systematically investigated.

Importantly, no existing study has comprehensively analyzed paired PF and serum samples to delineate both systemic and local metabolic alterations underlying graft restenosis. Furthermore, most predictive models in this field lack external validation and rarely demonstrate clear clinical utility [10]. Addressing these gaps is critical for advancing mechanistic understanding and improving long-term risk stratification.

Building upon our previous PF metabolomic research, we conducted an observational cohort study with targeted metabolomic profiling and secondary endpoint analysis in patients undergoing CABG. Paired PF and serum samples were systematically collected, and participants were followed for three years to assess long-term graft restenosis. We aimed to compare the prognostic value of metabolomic signatures derived from distinct biological compartments and to determine whether integrating PF and serum profiles enhances predictive performance. We hypothesized that PF, as a biofluid directly reflecting the local cardiac and vascular microenvironment, would capture unique metabolic dysregulations associated with vascular remodeling and restenosis, and that combining PF- and serum-based metabolomic signatures would substantially improve long-term risk prediction beyond traditional clinical models or single-biofluid approaches. Additionally, we explored metabolic pathways enriched by differential metabolites (DMs) in PF and serum to identify potential therapeutic targets.

In this study, we identified DMs in both PF and serum that were independently associated with long-term graft restenosis after CABG and systematically characterized the key metabolic pathways and their potential biological interactions. Based on these findings, we developed and validated a metabolite-based risk prediction model for long-term restenosis. Predictive performance and clinical net benefit were evaluated using Kaplan–Meier survival analysis, receiver operating characteristic (ROC) curves, Net Reclassification Improvement (NRI), Integrated Discrimination Improvement (IDI), and decision curve analysis (DCA). The model outperformed conventional clinical risk models, demonstrating superior predictive accuracy and meaningful clinical utility.

## 2. Materials and Methods

The study was approved by the Ethics Committee of Beijing Anzhen Hospital (approval number: 2019360x) and was conducted in accordance with the Declaration of Helsinki. Informed consent was obtained from all participants prior to surgery.

### 2.1. Participants and Study Design

Patients who underwent isolated coronary artery bypass grafting (CABG) at Beijing Anzhen Hospital between January 2020 and December 2023 were prospectively invited to participate in this study. Exclusion criteria included: history of documented arrhythmia, valvular heart disease, malignant tumor, thyroid dysfunction, requirement for redo or emergency CABG, concurrent cardiac procedures, incomplete clinical or follow-up data, or refusal to participate. The selection of clinical variables included in the present study was based on previously published literature investigating predictors of graft restenosis after CABG [11,12]. In total, 106 patients were enrolled in the discovery cohort and an additional 92 patients were enrolled in the validation cohort.

**Figure 1:**
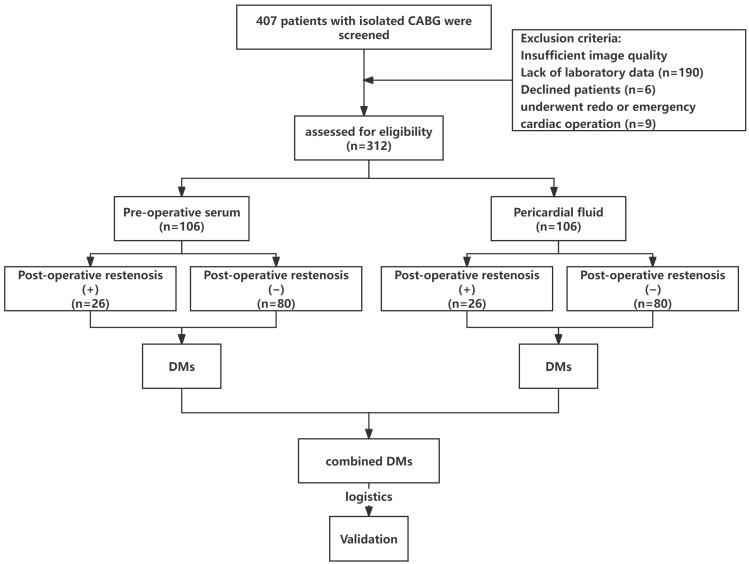
Flowchart of the study design.

Postoperative graft restenosis was defined as ≥50% luminal narrowing of the bypass graft, as determined by invasive coronary angiography according to widely accepted criteria [13,14]. Coronary angiograms were reviewed in a standardized manner by two experienced interventional cardiologists who were blinded to the clinical and metabolomics data. Discrepancies between observers were resolved by consensus or, when necessary, adjudicated by a third senior cardiologist. Quantitative assessment of percent diameter stenosis was performed visually, and measurements were made using consistent angiographic projection angles and calibrated catheter size as reference. Angiographic images were obtained during scheduled follow-up or in response to clinical symptoms suggestive of ischemia, and all readings adhered to a prespecified imaging interpretation protocol to ensure reproducibility. Patients were classified into restenosis and non-restenosis groups accordingly (Figure 1).

### 2.2. Clinical Laboratory Test and Sample Collection

Preoperative fasting peripheral venous blood was collected from all patients and centrifuged to isolate serum, which was then stored at −80 °C until assay. Pericardial fluid (PF) was collected intraoperatively immediately after opening the pericardial sac, centrifuged, and similarly stored at −80 °C for later analysis.

Demographic and clinical characteristics including age, sex, body mass index (BMI), history of hypertension, history of diabetes mellitus, operation time, number of coronary grafts, European System for Cardiac Operative Risk Evaluation (EuroSCORE) [15], left ventricular ejection fraction (LVEF), and left atrial enlargement were recorded for each participant. Hypertension was defined as baseline blood pressure ≥ 140/90 mmHg or use of antihypertensive medication [16], and diabetes was defined as fasting glucose ≥ 7.0 mmol/L or use of antidiabetic therapy [17]. Serum biochemical parameters including triglyceride (TG), total cholesterol (TC), high-density lipoprotein cholesterol (HDL-C), low-density lipoprotein cholesterol (LDL-C), small dense LDL-C (sdLDL-C), lipoprotein(a) [LP(a)], thyroid stimulating hormone (TSH), free triiodothyronine (fT3), and free thyroxine (fT4) were measured using an automated biochemistry analyzer and immunoassay system (Beckman Coulter, Brea, CA, USA).

### 2.3. Echocardiography

All patients underwent transthoracic echocardiography performed by experienced sonographers following a standardized protocol. LVEF was calculated using Simpson’s biplane method. Left atrial enlargement was defined as a left atrial diameter >40 mm in males or >38 mm in females [18].

### 2.4. UPLC-Qtrap/MS Analysis

Targeted metabolomic profiling was performed on PF and serum samples using an ultra-performance liquid chromatography–triple quadrupole/mass spectrometry (UPLC-Qtrap/MS) platform (AB Sciex QTRAP 6500+, Marlborough, MA, USA). A total of 50 μL of each thawed sample was mixed with 250 μL of 20% acetonitrile/methanol, vortexed for 3 min, and centrifuged at 12,000 rpm for 10 min at 4 °C. The supernatant was transferred, cooled at −20 °C for 30 min, and centrifuged again. A total of 180 μL of the final supernatant was transferred for analysis.

Chromatographic separation was achieved on an ACQUITY UPLC HSS T3 C18 column (2.1 × 100 mm, 1.8 μm) with a mobile phase of water containing 0.05% formic acid (A) and acetonitrile with 0.05% formic acid (B). The flow rate was 0.35 mL/min, and the column temperature was set at 40 °C. Both positive and negative ionization modes were used. Data acquisition was performed using Analyst 1.6.3 software, and metabolite quantification was performed using MultiQuant 3.0.3 software.

### 2.5. Metabolic Data Processing and Biomarker Selection

The quantitative data were Pareto scaled prior to multivariate statistical analysis. Principal component analysis (PCA) and orthogonal partial least squares discriminant analysis (OPLS-DA) were conducted using SIMCA-P software (version 14.0, Umetrics). Differential metabolites (DMs) were identified based on the S-plot of OPLS-DA combined with Mann–Whitney U test, with false discovery rate (FDR)-adjusted *p*-values < 0.05.

Metabolic pathway enrichment analysis was conducted using MetaboAnalyst (http://www.metaboanalyst.ca/, accessed on 20 August 2025). Logistic regression-based receiver operating characteristic (ROC) curve analyses were used to evaluate the discriminative ability of clinical and metabolomic features.

### 2.6. Statistical Analysis

Categorical variables were expressed as numbers and percentages, and compared using the chi-square or Fisher’s exact test. Continuous variables were presented as mean ± standard deviation or median (interquartile range) based on data distribution. The Kolmogorov–Smirnov test was used to assess normality. Student’s *t*-test or Mann–Whitney U test was used for continuous variables, as appropriate.

Univariate and multivariate logistic regression analyses were performed to determine independent predictors of restenosis. ROC curve analysis and area under the curve (AUC) values were calculated to assess the predictive performance of clinical, metabolomic, and combined models. Decision curve analysis (DCA) was used to evaluate the net clinical benefit. All analyses were conducted using R software (version 4.4.1), and two-tailed *p*-values < 0.05 were considered statistically significant.

Kaplan–Meier (KM) survival analysis was performed to evaluate restenosis-free survival among patients stratified by different risk groups. The follow-up duration was three years after CABG, and survival time was defined as the interval from surgery to the occurrence of graft restenosis or the last follow-up, whichever came first. Graft restenosis was defined as the event of interest (event = 1), while patients without restenosis or lost to follow-up were censored (event = 0). Survival curves were estimated using the survival package with the survfit() function, and differences between groups were compared using the log-rank test implemented via the survdiff() function. All survival analyses and graphical visualizations were conducted using R software (version 4.4.1), and a two-sided *p* < 0.05 was considered statistically significant.

## 3. Results

### 3.1. The Association Between Clinical Baseline Characteristics and Post-Operative Restenosis

A total of 106 individuals participated in the study (Figure 1). As shown in Table 1, no significant differences were observed in gender, BMI, graft materials, long-term illnesses (hypertension and diabetes mellitus), LVEF, duration of surgery, number of coronary grafts, and clinical lipid profiles (e.g., TG, HDL-C, LDL-C, and sdLDL-C between isolated CABG patients with and without long-term restenosis.

### 3.2. Univariate and Multivariate Analysis of Clinical Predictors for Restenosis

**Figure 2:**
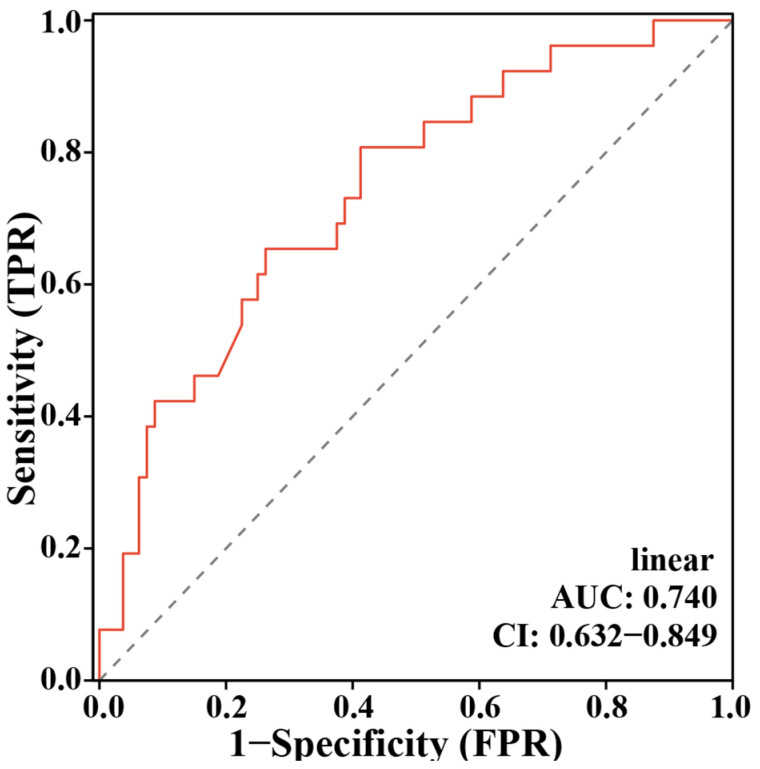
Receiver Operating Characteristic (ROC) curve for the clinical predictive model of post-operative graft restenosis. The model incorporated BMI, lipoprotein(a) [LP(a)], and free thyroxine (FT4) as independent predictors.

We performed multivariate Logistic regression analyses to explore the correlations between these baseline characteristics and restenosis. All clinical parameters were included in the model, and we found that BMI, LP(a), and FT4 remained significantly negatively correlated with restenosis (Table 2). Furthermore, a Cox regression-based ROC model incorporating BMI, LP(a), and FT4 yielded an unsatisfactory AUC = 0.740 (CI = 0.632–0.849) for identifying long-term postoperative restenosis in patients undergoing CABG (Figure 2).

### 3.3. Metabolite Signatures of PF and Serum in Patients with and Without Restenosis

**Figure 3:**
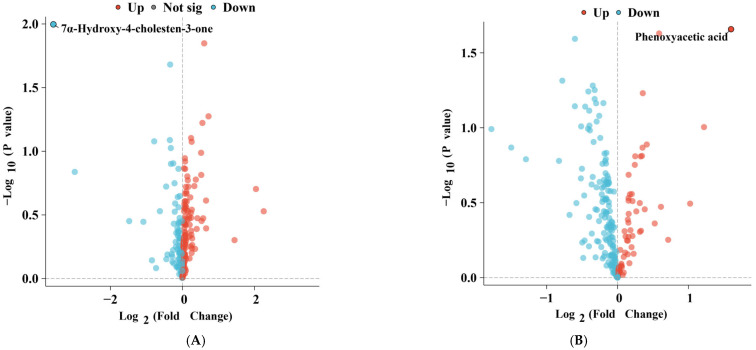
Volcano Plots of Differential Metabolite Analysis in Serum (**A**) and Pericardial Fluid (**B**) Between Restenosis and Non-restenosis Patients.

A UPLC-Qtrap/MS-based targeted metabolomic analysis was performed on PF and serum samples from patients with and without restenosis to quantify 516 metabolites, including a variety of hydrophilic and hydrophobic metabolite species. To characterize the DMs, differential analysis was conducted. As shown in Figure 3A,B, significant differences in the distribution of bile acid compounds (7α-Hydroxy-4-cholesten-3-one) and organic acids and their derivatives (Phenoxyacetic acid) were observed in PF and serum samples between patients with and without restenosis. Specifically, 7α-Hydroxy-4-cholesten-3-one (7αC4) levels were markedly decreased in the serum and PF of restenosis patients, while Phenoxyacetic acid (PAA) levels were significantly elevated in their PF.

### 3.4. Metabolic Pathway Analysis

**Figure 4:**
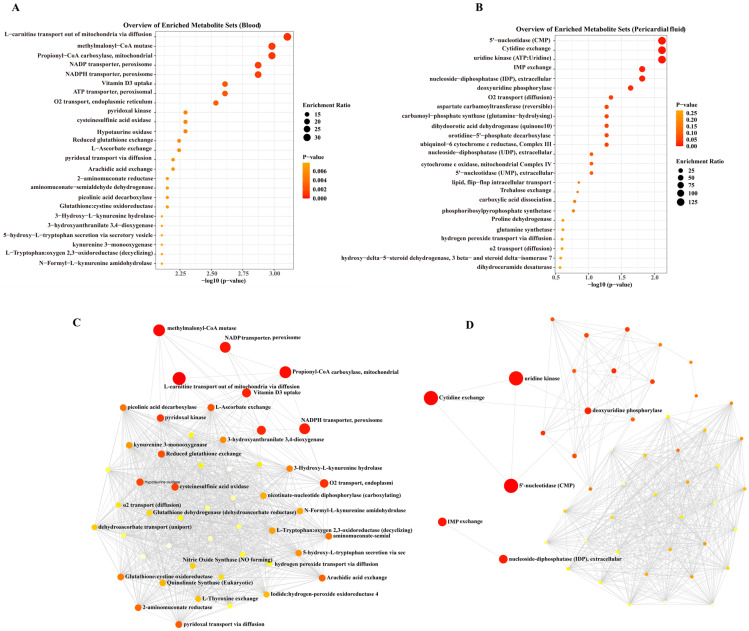
Pathway enrichment analysis of differentiated metabolites in Pericardial fluid (PF) and serum. (**A**) Serum-specific metabolic signatures. (**B**) PF-specific metabolic signatures. (**C**,**D**) Cross-talk between enriched pathways. Interaction networks reveal stronger functional connectivity among PF-enriched pathways (**D**) compared to serum-derived pathways (**C**). The size of the circles represents the pathway impact, while the color indicates statistical significance (with deeper red corresponding to lower *p*-values).

To identify key enriched metabolic pathways, we performed MetaboAnalyst-based pathway enrichment analysis on the differentiated metabolites in PF and serum. As shown in Figure 4A, tryptophan metabolism, propionate metabolism, and cysteine metabolism pathways (e.g., L-carnitine transport out of mitochondria via diffusion, Methylmalonyl-CoA mutase, and Propionyl-CoA carboxylase, mitochondrial) were significantly enriched in serum signatures that differentiated restenosis from non-restenosis. In regard to the signatures in PF (Figure 4B), pyrimidine nucleotide metabolism pathways (such as 5′-nucleotidase, Cytidine exchange, Uridine kinase, and Deoxyuridine phosphorylase) were notably enriched. Additionally, several lipid metabolism-related pathways were enriched in both PF and serum signatures, including Arachidic acid exchange. The interaction network among the enriched metabolic pathways is depicted in Figure 4C,D. The resultant plots demonstrated that the enriched metabolic pathways in PF exhibited stronger interactions with each other than those in serum, indicating strongly coordinated effects for the metabolic changes in the PF microenvironment.

### 3.5. Multi-Metabolites Model for the Prediction of Restenosis in Isolated CABG Patients

**Figure 5:**
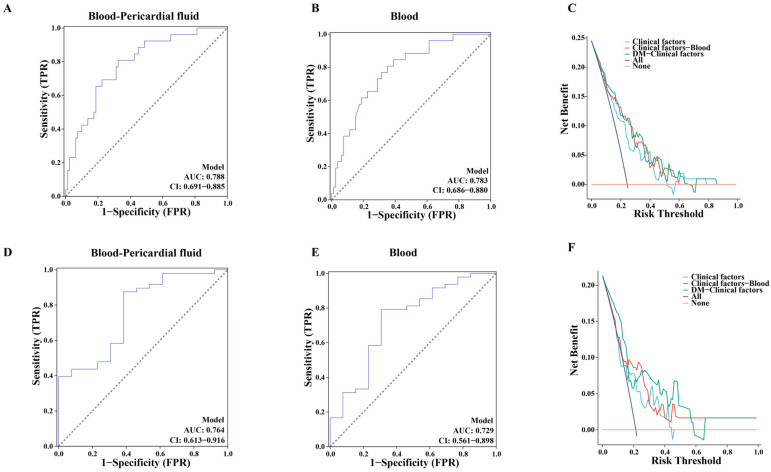
Predictive performance of 7α-Hydroxy-4-cholesten-3-one and Phenoxyacetic acid for restenosis risk. (**A**) Model comparison in training cohort. The combined model integrating clinical variables with serum and PF metabolomic features (7α-Hydroxy-4-cholesten-3-one and Phenoxyacetic acid). (**B**) The combined model integrating clinical variables with serum metabolomics in training cohort. (**C**) Decision curve analysis in training cohort. (**D**) Validation in independent cohort. ROC analysis of the two-metabolite model in 92 isolated CABG patients. (**E**) The combined model integrating clinical variables with serum metabolomics in validation cohort. (**F**) DCA curves further. Dashed line represents the ROC curve for a random guess.

Through the above analyses, we identified some potentially important DMs and enriched metabolic pathways in both PF and serum samples, suggesting that these metabolites and pathways may play an essential role in the development of restenosis. Therefore, we selected two metabolites, 7αC4 and PAA, for predictive model construction. A logistic regression model was developed by integrating clinical features with these two metabolites. As shown in Figure 5A, the combined model exhibited a higher classification accuracy for distinguishing patients with and without restenosis (AUC = 0.788, CI = 0.691–0.885) compared to the model using only clinical variables (AUC = 0.740, CI = 0.632–0.849, Figure 2) or the model using only serum metabolomic features (AUC = 0.783, CI = 0.686–0.880; Figure 5B). Furthermore, a DCA was performed to evaluate the clinical utility and net benefit of the new predictive model (Figure 5C). As shown in Figure 5C, the DM-clinical model obtains more clinical benefits than other models.

In Table 3, compared with the clinical model alone, the addition of the 7αC4 and Phenoxyacetic acid (PAA) has a significant increase in the predictive effect for predicting restenosis in terms of NRI (10% improvement, *p*  < 0.001) and IDI (2.94% improvement, *p*  =  0.00593).

**Figure 6:**
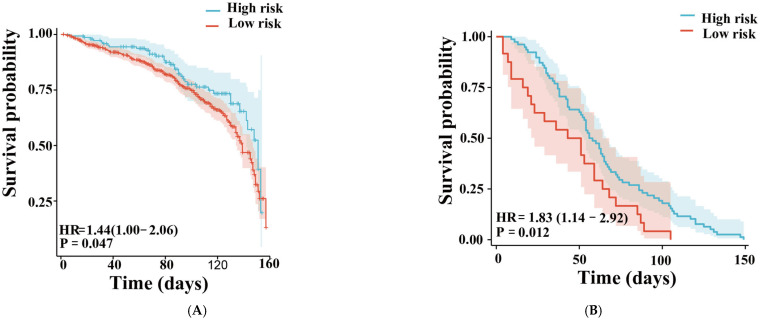
Survival curves. Training set on the left (**A**), validation set on the right (**B**).

Then, we used Cox regression analyses to investigate the correlation between the 2 metabolites and stenosis events. The KM-survival plots indicated that all 2 metabolites were significantly associated with stenosis (Figure 6A).

### 3.6. Validation of the Multi-Metabolites Model for Predicting Restenosis in an Additional Independent Cohort

The above results demonstrated that 7αC4 and PAA in both PF and serum were significantly different between patients with and without restenosis, and the combination of these two metabolic molecules in PF and serum exhibited great potential to predict the risk of new-onset restenosis. For convenience in clinical practice, serum samples from an additional independent cohort of 92 patients undergoing isolated CABG surgery were included as the validation set. Table 4 presents the demographic and clinical characteristics of the participants. Subsequently, we performed ROC analysis to investigate the association between these two metabolites and restenosis events. The results revealed that the combination of the two metabolites significantly improved the predictive accuracy for restenosis occurrence. The two-metabolite ROC model showed a good classification performance in differentiating patients with and without restenosis (AUC = 0.764, CI = 0.613–0.916; Figure 5D), which was notably superior to the models using a single metabolite alone. Notably, DCA curves further confirmed that the combined two-metabolite model offered substantial clinical benefit over the clinical model alone (Figure 5F). The KM-survival plots also indicated that the new combined model was significantly associated with stenosis (Figure 6B).

## 4. Discussion

This study, for the first time, integrates targeted metabolomic profiling of both pericardial fluid (PF) and serum to identify biomarkers with significant predictive value for long-term graft restenosis following CABG. Notably, PF—serving as a dynamic window into the local cardiac microenvironment—may reflect vascular remodeling processes earlier and more directly than systemic circulation [19,20].

We observed a compartment-specific pattern of metabolic dysregulation: a marked reduction of 7α-hydroxy-4-cholesten-3-one (7αC4) in serum contrasted sharply with abnormal accumulation of Phenoxyacetic acid (PAA) in PF. 7αC4 is a key intermediate in hepatic bile acid synthesis from cholesterol and serves as a sensitive marker of bile acid synthetic activity [21,22]. Circulating 7αC4 levels exhibit diurnal variation in parallel with bile acid production rhythms [23]. Beyond its established role in hepatobiliary physiology, emerging evidence links dysregulation of bile acid metabolism to cardiometabolic disorders. As a functional readout of the fibroblast growth factor 19 (FGF19)–bile acid feedback axis, altered 7αC4 levels have been associated with hypercholesterolemia, metabolic syndrome, and atherosclerosis [24].

Mechanistically, bile acid signaling through receptors such as farnesoid X receptor (FXR) and Takeda G protein-coupled receptor 5 (TGR5) modulates lipid metabolism, glucose homeostasis, inflammation, and vascular function. Perturbations in this pathway may therefore contribute to endothelial dysfunction and vascular remodeling. Additionally, fluctuations in 7αC4 under conditions of hepatic fibrosis or systemic inflammation have been associated with intestinal barrier disruption and endotoxin translocation, which may further promote vascular inflammation and plaque instability [25,26]. Pharmacological interventions that modulate bile acid synthesis and restore enterohepatic feedback have demonstrated improvements in lipid profiles and inflammatory status, indirectly supporting the cardiometabolic relevance of this pathway [27]. Collectively, these findings suggest that reduced serum 7αC4 may reflect impaired bile acid-mediated metabolic regulation associated with restenosis risk.

In contrast, PAA—commonly recognized as a component of herbicides but also detected as an exogenous human metabolite [27,28,29]—was markedly elevated in PF. Growing evidence suggests that PAA and its derivatives interact with lipid metabolism and platelet biology. Experimental studies indicate that certain PAA derivatives reduce total cholesterol (TC) and LDL-C without significantly affecting HDL-C, and may decrease triglyceride levels in hyperlipidemic models [30]. Moreover, specific PAA derivatives exhibit antiplatelet and antithrombotic properties. For example, BM 13.177 (4-[2-(benzenesulfonamido)-ethyl] phenoxyacetic acid) functions as a selective thromboxane A_2_ (TXA_2_) and prostaglandin endoperoxide antagonist, inhibiting secondary platelet aggregation and serotonin release [31]. The enrichment of PAA in PF may therefore reflect localized alterations in platelet activation or thrombo-inflammatory signaling within the graft microenvironment.

Further pathway analysis revealed that PF and serum metabolites converge on a synergistic imbalance of redox homeostasis and immune–metabolic reprogramming. In serum, activation of the tryptophan–kynurenine pathway was evidenced by a 3.2-fold elevation of quinolinic acid (*p* < 0.001), a metabolite capable of inducing vascular smooth muscle calcium overload through N-methyl-D-aspartate (NMDA) receptor activation [32,33]. Concurrently, uridine accumulation in PF may promote neutrophil extracellular trap (NET) formation via paracrine signaling. Together with elevated methylmalonic acid (MMA) in serum—a propionate-derived metabolite implicated in mitochondrial dysfunction—these alterations may amplify vascular injury and inflammatory remodeling [34].

This cross-compartment metabolic interaction suggests a coordinated process in which local PF perturbations initiate microenvironmental triggers that are subsequently reinforced by systemic metabolic amplification. Such metabolic crosstalk provides novel mechanistic insights into the pathogenesis of graft restenosis and highlights the importance of integrating local and systemic metabolic signatures in risk prediction.

From a translational standpoint, our 7αC4–PAA dual-biomarker model demonstrates three major advantages: enhanced predictive performance (AUC = 0.788 vs. clinical model 0.740, a 6.5% increase); the potential to provide early risk stratification using pre-operative samples; and the introduction of a new paradigm for metabolic intervention prior to the onset of restenosis. Importantly, this model directly suggests feasible therapeutic strategies. In the clinical setting, 7αC4 is commonly used as a diagnostic tool or metabolic biomarker. For instance, it is used to assess BAM or BAD—elevated serum levels of 7αC4 are indicative of increased hepatic bile acid synthesis secondary to impaired intestinal reabsorption of bile acids. In parallel, PAA toxicity could be addressed through screening for pesticide residues in serum and employing mitochondrial-targeted antioxidants such as MitoQ to mitigate damage [31,35]. More importantly, in our previous study on the impact of PF metabolomics on postoperative atrial fibrillation after CABG, we similarly found that the predominant functional pathways of pericardial fluid metabolites were related to lipid metabolism. This observation suggests that lipid metabolic processes are indeed highly active following CABG and may play a key role in the regulation of postoperative complications.

However, several limitations warrant consideration. The intraoperative PF samples captured only immediate postoperative metabolic states; hence, non-invasive, dynamic monitoring strategies are needed. Additionally, this study focused specifically on graft restenosis after CABG and did not integrate multiple other postoperative complications into a unified analysis, which may limit the generalizability of our findings to the broader spectrum of CABG-related adverse events. Furthermore, our study was conducted at a single center, and the sample size may not fully represent the diverse clinical and demographic characteristics seen in wider practice. Therefore, multicenter studies with larger cohorts and comprehensive postoperative outcome profiling are required to validate and extend these findings.

## 5. Conclusions

Looking ahead, we propose a metabolically targeted restenosis prevention strategy: utilizing preoperative 7αC4–PAA risk assessments to identify high-risk patients. This strategy not only provides a quantifiable tool for CABG postoperative management but also opens a new translational pathway for modulating vascular remodeling through the metabolic microenvironment. In future work, we will further investigate the comprehensive role of pericardial fluid metabolites in multiple postoperative complications following CABG, aiming to elucidate shared and distinct metabolic signatures that contribute to adverse outcomes. Such efforts may ultimately facilitate a more integrated, metabolism-based approach to risk stratification and personalized intervention in patients undergoing CABG.

## Figures and Tables

**Table 1:** Baseline Characteristics of Patients With and Without Post-operative Restenosis.

Characteristics	Non-Restenosis	Restenosis	*p* Value
*N*	80	26	
Age, mean ± sd	63.625 ± 8.0856	64.192 ± 7.0201	0.749
Gender, *n* (%)			0.562
Male	60 (56.6%)	18 (17%)	
Female	20 (18.9%)	8 (7.5%)	
hypertension, *n* (%)			0.660
Yes	47 (44.3%)	14 (13.2%)	
No	33 (31.1%)	12 (11.3%)	
diabetes, *n* (%)			0.433
Yes	30 (28.3%)	12 (11.3%)	
No	50 (47.2%)	14 (13.2%)	
BMI, mean ± sd	25.899 ± 3.5996	25.239 ± 2.2415	0.273
Euro score, median (IQR)	5 (4, 7)	6 (5, 6)	0.755
Operation duration, median (IQR)	4 (4, 5)	4 (4, 5)	0.877
The number of Bridges built, median (IQR)	3 (3, 4)	4 (3, 4)	0.269
Whether the left atrium is enlarged, *n* (%)			0.487
No	43 (40.6%)	16 (15.1%)	
Yes	37 (34.9%)	10 (9.4%)	
Bridge-building organization, *n* (%)			0.395
vein	62 (58.5%)	18 (17%)	
artery	18 (17%)	8 (7.5%)	
Ejection fraction, median (IQR)	60.5 (55, 66)	60.5 (55, 65)	0.617
TG, median (IQR)	1.385 (1.0475, 2.08)	1.37 (1.1125, 1.81)	0.834
TC, median (IQR)	3.85 (3.3575, 4.435)	3.895 (3.27, 4.52)	0.994
HDL-C, median (IQR)	0.97 (0.8275, 1.0725)	1.03 (0.89, 1.15)	0.133
LDL-C, median (IQR)	2.025 (1.72, 2.5375)	2.11 (1.5475, 2.5325)	0.814
sd-LDL, median (IQR)	0.58 (0.4, 0.84)	0.555 (0.3575, 0.885)	0.986
LP(a), median (IQR)	35.2 (9.5, 89.7)	42.4 (24.45, 139.12)	0.167
TSH, median (IQR)	1.843 (1.124, 2.966)	1.572 (1.0815, 3.0965)	0.991
FT4, mean ± sd	11.701 ± 1.9509	12.692 ± 1.8912	0.053

**Table 2:** Univariate and Multivariate Logistic Regression Analyses of Clinical Characteristics Associated With Post-operative Restenosis.

Characteristics	Total (*N*)	Univariate Analysis		Multivariate Analysis	
		Odds Ratio (95% CI)	*p* Value	Odds Ratio (95% CI)	*p* Value
Gender	106				
Male	78	Reference			
Female	28	1.333 (0.503–3.533)	0.563		
Age	106				
≥60	35	Reference			
<60	71	1.146 (0.442–2.973)	0.779		
BMI	106				
BMI ≥ 28	54	Reference		Reference	
24 ≤ BMI ≤ 27.9	27	0.571 (0.196–1.665)	0.305	0.391 (0.122–1.258)	0.115
18.5 ≤ BMI ≤ 23.9	22	0.200 (0.042–0.952)	0.043	0.174 (0.034–0.894)	0.036
BMI < 18.5	3	0.000 (0.000–Inf)	0.991	0.000 (0.000–Inf)	0.991
hypertension	106				
Yes	61	Reference			
No	45	1.221 (0.501–2.974)	0.661		
Diabetes	106				
Yes	42	Reference			
No	64	0.700 (0.286–1.712)	0.434		
Euro score	106	1.033 (0.832–1.283)	0.768		
Operation duration	106	1.037 (0.552–1.947)	0.911		
The number of Bridges built	106	1.221 (0.694–2.148)	0.489		
Postoperative hospital stay duration	106	0.999 (0.903–1.106)	0.990		
Whether the left atrium is enlarged	106				
No	59	Reference			
Yes	47	0.726 (0.294–1.794)	0.488		
Ejection fraction	106				
≥50%	94	Reference			
<50%	12	1.636 (0.450–5.955)	0.455		
TG	106	0.884 (0.498–1.572)	0.676		
LDL-C	106	0.936 (0.525–1.670)	0.824		
sd-LDL	106	1.063 (0.268–4.226)	0.931		
LP(a)	106	1.005 (0.999–1.011)	0.084	1.007 (1.000–1.013)	0.044
TSH	106	0.957 (0.727–1.261)	0.755		
FT3	106	0.505 (0.177–1.443)	0.202		
FT4	106	1.270 (0.980–1.645)	0.071	1.343 (1.019–1.769)	0.036

**Table 3:** Predicted value of new model compared to clinical model.

	Categorical NRI (95% CI)	Continuous NRI (95% CI)	IDI (95% CI)
New model vs. Clinical model	0.1 (0.0343 ~ 0.1657) *p* = 0.00287	0.1962 (−0.0678 ~ 0.4602) *p* = 0.14531	0.0294 (0.0085 ~ 0.0504) *p* = 0.00593

Continuous NRI was chosen for the primary analysis and categorical for the ancillary analysis. Statistical significance was set at the 2-tailed 0.05 level without multiplicity adjustment. Abbreviations: CI, confidence interval; NRI, net reclassification index; IDI, integrated discrimination improvement. supported the clinical advantage of the two-metabolite model.

**Table 4:** Baseline Characteristics of the Validation Cohort Stratified by Restenosis Status.

Characteristics	Restenosis	Non-Restenosis	*p* Value
*N*	22	70	
Age, mean ± sd	61.727 ± 8.4863	63.529 ± 6.4352	0.367
Gender, *n* (%)			0.990
Female	6 (6.5%)	19 (20.7%)	
Male	16 (17.4%)	51 (55.4%)	
hypertension, *n* (%)			0.566
Yes	12 (13%)	43 (46.7%)	
No	10 (10.9%)	27 (29.3%)	
diabetes, *n* (%)			0.412
Yes	10 (10.9%)	25 (27.2%)	
No	12 (13%)	45 (48.9%)	
BMI, mean ± sd	25.533 ± 3.1528	25.492 ± 2.6108	0.951
Euro score, median (IQR)	5 (4, 6.75)	5 (4.25, 7)	0.535
Operation duration, median (IQR)	4 (4, 5)	4 (4, 4.375)	0.295
The number of Bridges built, *n* (%)			0.790
3	9 (9.8%)	33 (35.9%)	
4	12 (13%)	32 (34.8%)	
2	1 (1.1%)	3 (3.3%)	
5	0 (0%)	2 (2.2%)	
Whether the left atrium is enlarged, *n* (%)			0.872
0	15 (16.3%)	49 (53.3%)	
1	7 (7.6%)	21 (22.8%)	
Ejection fraction, median (IQR)	62 (54.25, 65)	62 (58.25, 65)	0.607
TG, median (IQR)	1.405 (1.12, 1.66)	1.525 (1.135, 1.93)	0.423
TC, mean ± sd	4.0286 ± 0.84002	4.2547 ± 0.94628	0.319
HDL-C, median (IQR)	0.945 (0.8175, 1.145)	0.985 (0.8725, 1.13)	0.749
LDL-C, mean ± sd	2.2323 ± 0.64464	2.341 ± 0.73997	0.538
sd-LDL, median (IQR)	0.54 (0.47, 0.76)	0.6 (0.4575, 0.8325)	0.850
LP(a), median (IQR)	37.2 (22.7, 73)	49.3 (15.6, 149.32)	0.845
TSH, median (IQR)	1.028 (0.7085, 1.631)	2.1095 (1.4165, 4.364)	<0.001
FT3, median (IQR)	4.78 (4.615, 5.365)	4.71 (4.205, 4.925)	0.105
FT4, mean ± sd	11.962 ± 2.0707	11.354 ± 1.6876	0.242

## Data Availability

Data is contained within the article.

## Abbreviations

The following abbreviations are used in this manuscript:

CABG	Coronary Artery Bypass Grafting
PF	Pericardial Fluid
BMI	Body Mass Index
EuroSCORE	European System for Cardiac Operative Risk Evaluation
LVEF	Left Ventricular Ejection Fraction
TG	Triglyceride
TC	Total Cholesterol
HDL-C	High-Density Lipoprotein Cholesterol
LDL-C	Low-Density Lipoprotein Cholesterol
sdLDL-C	Small Dense Low-Density Lipoprotein Cholesterol
LP(a)	Lipoprotein(a)
TSH	Thyroid-Stimulating Hormone
fT3/FT3	Free Triiodothyronine
fT4/FT4	Free Thyroxine
UPLC-Qtrap/MS	Ultra-Performance Liquid Chromatography–Triple Quadrupole/Mass Spectrometry
PCA	Principal Component Analysis
OPLS-DA	Orthogonal Partial Least Squares Discriminant Analysis
FDR	False Discovery Rate
ROC	Receiver Operating Characteristic
AUC	Area Under the Curve
DCA	Decision Curve Analysis
PAA	Phenoxyacetic Acid
FXR	Farnesoid X Receptor
TGR5	Takeda G Protein-Coupled Bile Acid Receptor 5
NMDA	N-Methyl-D-Aspartate
NET	Neutrophil Extracellular Trap
MMA	Methylmalonic Acid
PET	Positron Emission Tomography
IDI	Integrated Discrimination Improvement
KM	Kaplan–Meier
NRI	Net Reclassification Improvement
DM	Differential Metabolite
7αC4	7α-Hydroxy-4-cholesten-3-one
BAM	Bile Acid Malabsorption
BAD	Bile Acid Diarrhea
NAFLD	Non-Alcoholic Fatty Liver Disease
TXA2	Thromboxane A_2_
